# Evaluating Hydroxyapatite, Gold Nanoparticles, and Graphene-Copper as Bimodal Agents for X-ray and Computed Tomography

**DOI:** 10.3390/bioengineering10020238

**Published:** 2023-02-10

**Authors:** Bruno Pugliese Pereira, Claudia Antoine, Aline Oliveira da Silva de Barros, Leonardo de Castro Pacífico, Martha Sahylí Ortega Pijeira, Alexandre Malta Rossi, Eduardo Ricci-Junior, Luciana Magalhães Rebelo Alencar, Ralph Santos-Oliveira

**Affiliations:** 1 Laboratory of Nanoradiopharmacy and Synthesis of Novel Radiopharmaceuticals, Nuclear Engineering Institute Brazilian Nuclear Energy Commission, Rio de Janeiro 21941906, RJ, Brazil; 2 Department of Radiological Sciences, Institute of Biology Roberto Alcântara Gomes State University of Rio de Janeiro, Rio de Janeiro 20550013, RJ, Brazil; 3 Department of Condensed Matter, Applied Physics and Nanoscience, Brazilian Center for Research in Physics, Rio de Janeiro 22290180, RJ, Brazil; 4 School of Pharmacy, Federal University of Rio de Janeiro, Rio de Janeiro 21941900, RJ, Brazil; 5 Biophysics and Nanosystems Laboratory, Department of Physics, Federal University of Maranhão, São Luis 65065690, MA, Brazil; 6 Laboratory of Radiopharmacy and Nanoradiopharmaceuticals, Rio de Janeiro State University, Rio de Janeiro 23070200, RJ, Brazil

**Keywords:** diagnosis, radiology, biomedical, medical, nanotechnology

## Abstract

A global need exists for new and more effective contrast agents for computed tomography and traditional X-ray modalities. Among the few options available nowadays, limitations imposed by industrial production, performance, and efficacy restrict the use and reduce the potential of both imaging techniques. The use of nanomaterials as new contrast agents for X-ray and computed tomography is an innovative and viable way to increase the options and enhance performance. In this study, we evaluated eight nanomaterials: hydroxyapatite doped with zinc (Zn-HA 10%); hydroxyapatite doped with strontium (Sr-HA 10%); hydroxyapatite without thermal treatment (HA 282 STT); thermally treated hydroxyapatite (HA 212 500 °C and HA 01.256 CTT 1000 °C); hydroxyapatite microspheres (HA microspheres); gold nanoparticles (AuNP); and graphene oxide doped with copper (Cu-GO). The results showed that for both imaging modalities; HA microspheres were the best option, followed by hydroxyapatite thermally treated at 1000 °C. The nanomaterials with the worst results were hydroxyapatite doped with zinc (Zn-HA 10%), and hydroxyapatite doped with strontium (Sr-HA 10%). Our data demonstrated the potential of using nanomaterials, especially HA microspheres, and hydroxyapatite with thermal treatment (HA 01.256 CTT 1000 °C) as contrast agents for X-ray and computed tomography.

## 1. Introduction

Medical imaging plays a key role in the early diagnosis, monitoring of medical treatments, and therapeutic response assessment [1,2]. Medical imaging enables the noninvasive visualization of the inside of the human body and provides anatomical, morphological, and functional information. Ultrasonography, X-ray imaging, computed tomography (CT), magnetic resonance imaging (MRI), single photon emission computed tomography (SPECT), and positron emission tomography (PET) are some of the medical imaging techniques commonly used today. Among these, X-ray imaging and CT are very popular worldwide. X-ray imaging is extensively used because it is fast and probably the most cost-effective medical imaging modality [3]. CT is also an X-ray-based technology with widespread use due to its high 3D spatial resolution of anatomic features, low cost, fast scan time, and good patient compatibility [4,5]. 

During imaging, X-rays cross through the patient and are attenuated by the tissue. This attenuation depends on the mass attenuation coefficient (μ/ρ; cm^2^/g) and the density (ρ; g/cm^3^) of all materials in that path [6]. While some tissues are easily detected, such as lungs and bones, X-ray imaging techniques struggle to distinguish soft tissues [7]. Conventional radiography is unable to visualize low-contrast tissues and structures due to, for instance, inefficient X-ray absorption, high scatter-to-primary X-ray ratios, superimposition and conspicuity [8]. Therefore, X-ray imaging techniques may require the administration of contrast agents to improve the contrast between tissue, fluid, and/or anatomical structures [9]. The contrast agents commonly reported contain elements with a high atomic number (Z) because the attenuation of X-rays is dependent on Z raised to the third power [9,10]. Iodine (Z = 53), tantalum (Z  =  73), rhenium (Z = 75), gold (Z = 79), and bismuth (Z = 83) are some examples of high-Z elements that have been evaluated as contrast agents [6,9,11,12,13]. Based on the combination of cost, availability, toxicity, and imaging performance, iodine is the only element approved among them for intravascular administration for CT and X-ray imaging [6]. However, several symptoms, such as headache, itching, nausea, vomiting, fever, skin rash, and musculoskeletal pain, have been reported by some patients as delayed adverse reactions 1 h to 1 week after intravascular iodinated agent injection [14]. In addition, other aspects related to iodine-based contrast agents are also concerns, such as the possibility of anaphylactic shock [15], and the need to repeatedly use large doses to counteract rapid renal excretion to obtain a good contrast [9]. 

Therefore, many works have been focused on discovering new contrast agents that are more suitable for X-ray/CT scans, mostly using nanoparticles due to their very attractive properties. Nanosized particles have a larger surface-to-volume ratio and longer circulation time than microparticles [16], enabling image acquisition at delayed time points after administration of low doses. In addition, nanoparticles can be easily surface-functionalized and prepared with different shapes and sizes to mediate the targeting ability, distribution, elimination, and toxicity [17,18]. Nanoparticles are able to passively accumulate, for instance, in solid tumors because of the enhanced permeability and retention (EPR) effect, locally generating X-ray images [19,20]. Gold [4,5,20], bismuth [21,22], tantalum [23,24], rhenium [25], silver [26], and other nanoparticles have been reported so far with promising outcomes as X-ray contrast agents. Among the metal core-based nanoparticles, gold has been highlighted due to its high X-ray attenuation coefficient, with a K-edge energy of 80.7 keV and density of 19.3 g/cm^3^, suitable biocompatibility, and well-known control of surface chemistry, shape, and size during production [4,5,7]. 

On the other hand, hydroxyapatite is a calcium phosphate ceramic widely used in biomedical applications due to its similar chemical composition to bone and teeth [27]. This ceramic biomaterial is biocompatible, bioactive, and thermodynamically stable in body fluids [28]. The flexible hexagonal structure of hydroxyapatite enables the replacement of the phosphate anions by other anionic complexes as well as the substitution of the calcium cations by many metal cations [29,30]. These favorable properties have expanded the biomedical applications of hydroxyapatite. For medical imaging, silver (Ag^+^) and gadolinium (Gd^3+^) ions co-substituted on hydroxyapatite nanoparticles have exhibited potential as bimodal contrast agents for CT and MRI [31]. In addition, doping of hydroxyapatite nanocrystals with europium (Eu^3+^) and gadolinium (Gd^3+^) ions resulted in tri-modal contrast in fluorescence imaging, MRI, and X-ray imaging [32]. 

Furthermore, graphene oxide (GO) is another promising candidate as a contrast agent for CT [26,33,34,35]. GO has a two-dimensional crystalline structure organized in a hexagonal sp2-carbon pattern with several oxygen-containing functionalities (hydroxyl, carboxyl, epoxide) on the surface [36]. These oxygen groups enable easy covalent functionalization to improve biocompatibility and reduce toxicity. GO also has strong thermal and electrical conductive properties as well as long-range π conjugation and the ability to form stable suspensions in water [34,36]. Previous works have reported the excellent potential as contrast agents for CT and X-ray imaging of graphene oxide/Ag nanoparticles [26,33,34], and reduced graphene nanoplatelets doped with iodine and manganese ions for bimodal CT/MRI imaging [35]. In addition, multifunctional stimuli-responsive 2D-based smart nanocomposites, comprising gold nanoparticles and superparamagnetic iron oxides scaffolded within graphene oxide nanosheets, were found to be promising candidates for multimodal CT/MRI image-guided cancer therapy [37].

In this study, we assessed hydroxyapatite nanoparticles (HA NP) produced using varied protocols, graphene oxide doped with copper (Cu-GO), and gold nanoparticles (AuNP) as contrast agents for X-ray and CT imaging.

## 2. Methodology

### 2.1. Production and Characterization of Nanoparticles

All the HA NP and Cu-GO nanoparticles were synthesized and characterized using previous works, as described below. The AuNP were acquired from Sigma Aldrich (São Paulo, Brazil) with the following characteristics: nanopowder, <100 nm particle size, and 99.9% trace metals basis. All the reagents and solvents were purchased from Sigma Aldrich (São Paulo, Brazil).

#### 2.1.1. Hydroxyapatite

Hydroxyapatite (HA) powders were precipitated by dropwise addition of a (NH_4_)_2_HPO_4_ aqueous solution containing NH_4_OH to a Ca(NO_3_)_2_ solution at 90 °C and pH of 11, following procedures of Resende et al. [38]. The precipitate was separated by filtration, repeatedly washed with boiling deionized water, and dried at 100 °C for 24 h. The dried powder was manually ground, and <210 µm particles were separated by sieving. HA powders from various syntheses were maintained without thermal treatment or calcined at 500 °C and 1000 °C, respectively. HA microspheres were prepared from a mixture of HA powder and the polymer alginate and extruded into a CaCl_2_ solution. The microspheres were dried overnight at 25 °C, and sintered and calcined at 1100 °C for 2 h. HA doped with zinc (ZnHA, 5% molar) or strontium (SrHA, 10% molar) was synthesized by adding (NH_4_)_2_HPO_4_ aqueous solution to a Ca(NO_3_)_2_ and Zn(NO_3_)_2_ or Sr(NO_3_)_2_ solution at 90 °C and pH of 9. Calcium and phosphorous concentration, crystalline phases, particle size, and morphology were determined by fluorescence of X-ray (FRX), Fourier transform infrared spectroscopy (FTIR), X-ray diffraction (XRD), and scanning electron microscopy (SEM).

#### 2.1.2. Hydroxyapatite Doped with Zinc (Zn-HA 10%)

Hydroxyapatite dopped with zinc (10%) was produced and fully characterized as described by Pedrosa et al. [39] and Martinez-Zelaya et al. [40]. Briefly, Zn-HA was precipitated through the dropwise addition of an aqueous (NH_4_)_2_HPO_4_ solution to a solution containing Zn(NO_3_)_2_ at a pH of 9.0, followed by stirring of the suspension for 3 h at 90 °C. The precipitate was then separated by filtration, repeatedly washed with boiling deionized water, and subsequently dried at 100 °C for 24 h. The characterization was performed by FTIR, atomic absorption spectroscopy (AAS), and XRD. 

#### 2.1.3. Hydroxyapatite Doped with Strontium (Sr-HA 10%)

Hydroxyapatite doped with strontium (10%) was produced and fully characterized as described by Terra et al. [41]. Briefly, strontium-doped HA (Sr-HA) produced by precipitation with an increasing ion exchange between calcium (Ca) and Sr-HA was precipitated through dropwise addition of an aqueous (NH4)_2_HPO_4_ solution to a solution containing Sr(NO_3_)_2_ at pH of 9.0, followed by stirring of the suspension for 3 h at 90 °C. The precipitate was then separated by filtration, repeatedly washed with boiling deionized water, and subsequently dried at 100 °C for 24 h. The powder was characterized by XRD, (FTIR), and extended X-ray absorption fine structure (EXAFS) spectroscopy.

#### 2.1.4. Hydroxyapatite (HA) without Thermic Treatment (HA 282 STT) and with Thermic Treatment at 500 °C (HA 212 500 °C) and 1000 °C (HA 01.256 CTT 1000 °C)

These three types of hydroxyapatite were produced and fully characterized as described by Albernaz et al. [42]. Briefly, hydroxyapatite was precipitated by dropwise addition of (NH_4_)_2_HPO_4_ aqueous solution containing NH_4_OH to a Ca(NO_3_)_2_ solution at 37 °C, and pH equal to 11. The precipitate was separated by filtration and repeatedly washed with deionized boiling water. Then, the HA without thermic treatment was dried at 100 °C for 24 h. On the other hand, the thermally treated HA was dried at 500 °C or 1000°C, respectively, for 24 h. Calcium and phosphorous concentrations (Ca/P = 1.66) were determined by X-ray fluorescence spectroscopy. Mineral phase and crystallinity were evaluated by XRD. Phosphate species and OH^−^ groups in apatite structure were identified by FTIR in transmission mode from 400 to 4000 cm^−1^. Crystalline size (D) along hydroxyapatite (002) and (300) directions was determined by the Debye–Sherrer formula (Equation (1)), where β is full width at half maximum of the diffraction peak (values in radians) and k = 0.9.

(1) $$D=\frac{k\lambda }{\beta \cos\theta }$$

#### 2.1.5. Microspheres of Hydroxyapatite (HA Microspheres)

The hydroxyapatite microspheres were produced and fully characterized as described by Martinez-Zelaya et al. [40]. Briefly, HA powder was gently dispersed in a 10 mg/mL aqueous solution of sodium alginate to achieve an alginate/powder ratio of 1:15 (6.7 wt% of alginate). The alginate/powder mixture was extruded dropwise at room temperature into a 0.15 M CaCl_2_ solution, using a needle with 0.70 mm diameter. Microspheres were formed instantaneously and were allowed to mature in the CaCl_2_ solution for 24 h for complete gelation. The microsphere was dried overnight at 25 °C and sintered and calcined at 1100 °C for 2 h. The authors characterized the microspheres by synchrotron radiation-based X-ray microtomography (SR-μCT) and SEM. 2.1.6 Graphene oxide doped with copper (Cu-GO).

#### 2.1.6. Graphene Oxide Doped with Copper (Cu-GO)

The graphene oxide doped with copper was produced and fully characterized following the method described by D Ni’maturrohmah et al. [43]. Briefly, 0.5 g of graphene oxide and 0.1 M of NaCl were diluted in 25 mL of deionized water, stirred for 20 min, followed by electrolysis using an electrochemical cell at 3 and 5 volts for 30 min at room temperature using a carbon rod as cathode and a copper plate as anode. Finally, the resulting product was dried at 100 °C for 1 h. The characterization was performed by X-ray powder diffraction and SEM.

### 2.2. Bimodal Imaging

#### 2.2.1. Computed Tomography

The CT attenuation properties of all the samples were evaluated using a Siemens SOMATOM Emotion scanner (CT 2014), and axial sections were analyzed. The parameters were as follows: mAs fixed: 40, kV: 80, pitch: 1.5 mm, slice: 5 mm (Acq. 16 × 0.6 mm), and reconstruction of 67 images (1.5 mm/1.5 mm), FOV 120 mm, acquisition time 5.18 s, with B60S medium sharp (lung parenchyma) filter reconstruction, lung window, craniocaudal direction, and increment of 1.5 mm.

#### 2.2.2. X-ray Imaging

The X-ray attenuation properties of all the samples were evaluated using a Siemens Polydoros LX 50 X-ray generator. The study was performed by the standard wrist technique (mAs 20, ms 71, and 46 Kv).

## 3. Results

**Figure 1:**
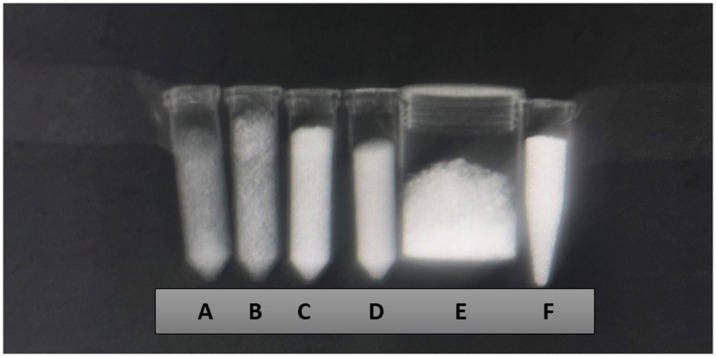
X-ray imaging using the wrist technique (mAs 20, ms 71, and 46 Kv) of the samples Zn-HA 10% (A), Sr-HA 10% (B), HA 01.256 CTT 1000 °C (C), HA microspheres (D), AuNP (E), and Cu-GO (F).

**Figure 2:**
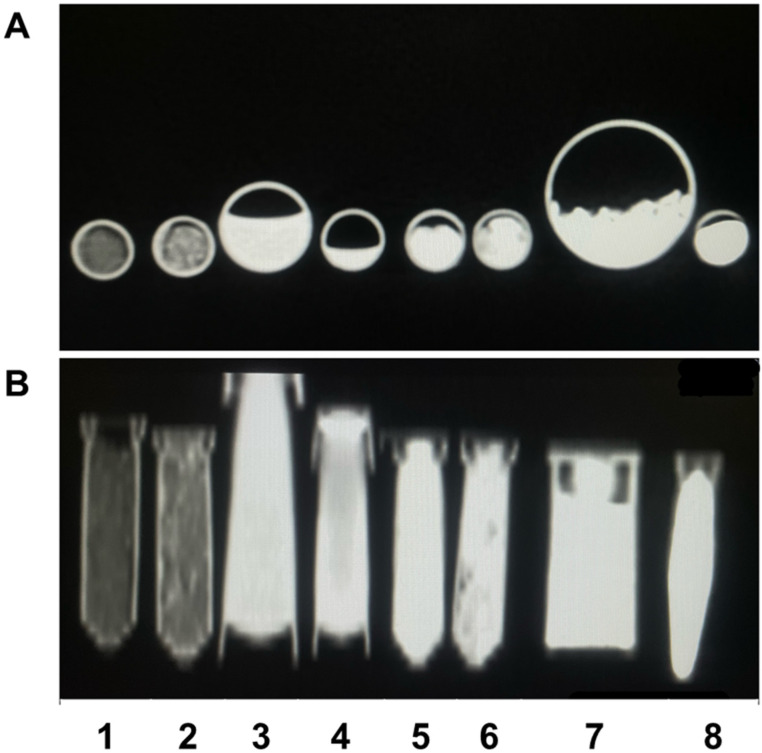
CT imaging showing axial section (**A**) and coronal section (**B**) of the samples Zn-HA 10% (1), Sr-HA 10% (2), HA 282 STT (3), HA 212 500 °C (4), HA 01.256 CTT 1000 °C (5), HA microspheres (6), AuNP (7), and Cu-GO (8).

Zn-HA 10%, Sr-HA 10%, HA 01.256 CTT 1000 °C, HA microspheres, AuNP, and Cu-GO samples exhibited a significant X-ray attenuation (Figure 1). In addition, the CT imaging (Figure 2) results demonstrated that all the samples evaluated are able to attenuate the X-ray and form a contrast.

The X-ray attenuation coefficient (µ) expressed in cm^−1^ and Hounsfield units for the eight samples are overviewed by Table 1. It possible to observe that the better agent for X-ray contrast is the hydroxyapatite microspheres followed by hydroxyapatite thermally treated at 1000 °C. The worst agent for contrast was hydroxyapatite doped with zinc, followed by hydroxyapatite doped with strontium.

## 4. Discussion

The resulting X-ray and CT image is a grayscale map that is directly related to the linear radiation attenuation coefficients within each material or tissue [7,44,45]. The attenuation coefficient is a measure used to estimate the absorption of radiation suffered by the X-ray beam when passing through an object. Thus, materials that absorb more photons have higher attenuation values and are displayed in white in the image, while materials or tissues that absorb fewer photons have lower attenuation values and are represented in black [44,45].

The computer is able to calculate the numerical value that represents the attenuation coefficient for each volume element (voxel). This value corresponds to the average amount of radiation absorption of a given tissue/material, represented by the pixel on the monitor [46,47]. After the calibration performed internally in the tomography, the density of the tomogram is adjusted to reference values such as pure water equal to 0 Hounsfield units (HU) and standardized air to −1000 HU. The relationship between the tissue/material attenuation coefficient and Hounsfield units is known as the Hounsfield Scale [46].

X-ray/CT contrast agents play a crucial role in distinguishing between tissues with low or similar attenuation coefficients. For example, soft tissues such as adipose tissue or soft materials consisting of hydrocarbon structures are not very sensitive to X-rays [47,48]. Thus, to improve image quality, contrast agents can be used. Contrast agents contain molecules that have a high attenuation coefficient and are expected to increase the attenuation of X-rays at the site of interest compared to surrounding tissues, improving image visualization [48,49,50,51].

In the present study, we evaluated hydroxyapatite doped with zinc (Zn-HA 10%) or strontium (Sr-HA 10%), hydroxyapatite without thermal treatment (HA 282 STT) or thermally treated at 500 °C (HA 212 500 °C) and 1000 °C (HA 01.256 CTT 1000 °C), HA microspheres, as well as graphene oxide doped with copper (Cu-GO), and AuNP as contrast agents for X-ray/CT scans. Among them, HA microspheres exhibited the highest contrast (2195 HU) and X-ray linear attenuation coefficient (0.764 cm^−1^). This result was similar to the contrast effect reported by Madhumathi et al. [31] for a hydroxyapatite sample co-substituted with 0.25 Ag and 0.75 Gd at. % substitution (2199 HU). These authors demonstrated that silver and gadolinium substitution further improved the contrast in CT scans compared with pure hydroxyapatite (1011 HU) [31].

On the other hand, hydroxyapatite doped with zinc (Zn-HA 10%) or strontium (Sr-HA 10%) displayed the lowest contrast and X-ray attenuation (−575 HU/0.102 cm^−1^ and −438 HU/0.134 cm^−1^, respectively). These negative HU values indicated the low radiodensity of the samples, and consequently, their lesser X-ray beam absorption, resulting in weaker contrast of the images [52]. In addition, the low contrast of both doped HA samples was expected due to the lower atomic numbers of zinc (Zn = 30) and strontium (Z = 38) than iodine (Z = 53), producing probably even lower HU than iodine [6].

Furthermore, the increase of temperature from 500 °C to 1000 °C enhanced the contrast in the CT between the thermally treated HA samples (HA 212 500°C: 168 HU/0.279 cm^−1^ vs. HA 01.256 CTT 1000 °C: 596 HU/0.381 cm^−1^). Conversely, the HA sample without thermal treatment produced higher contrast (HA 282 STT: 203 HU/0.288 cm^−1^) than the HA sample thermally treated at 500 °C. The HU values of these three HA nanocomposites were even greater than the HU values for both gold nanoparticles (AuNP: 34 HU/0.247 cm^−1^) and graphene oxide doped with copper (Cu-GO: 6 HU/0.240 cm^−1^). All the HA nanoparticles were imaged as dry powders, while both Cu-GO and AuNP were used in solution (2 mg/mL). Other works with gold nanoparticles with different shapes and symmetrical to anisotropic morphology have reported their markedly higher contrast (424–3378 HU) depending on the gold concentration (7.67–35.00 mg/mL) [53]. However, gold is an expensive metal, which could limit the application of AuNP as a contrast agent.

According to previous works, reduced graphene nanoplatelets doped with iodine (33.8 mM) and manganese ions displayed a radiodensity of 1980 HU [35]. Moreover, multifunctional stimuli-responsive 2D-based smart nanocomposites, comprising AuNP and superparamagnetic iron oxides scaffolded within GO nanosheets, showed an approximate CT contrast of 60 HU at a gold concentration of 2.5 mg/mL [37]. Regarding graphene oxide doped with copper as a contrast agent, we are unaware of any reports. Our results showed low contrast for GO-Cu (6 HU), but it was higher than the Zn/Sr-doped samples despite its slightly lower atomic number (Z = 29). Hence, the clearer contrast of the image of the sample GO-Cu was probably due to the chemical properties of GO itself. GO has a two-dimensional crystalline structure organized in a hexagonal sp2-carbon pattern with several oxygen-containing functionalities on the surface [36]. This might convert it into an electron-dense material, enhancing the X-ray attenuation.

Therefore, the HA microspheres presented the highest radiodensity (HU value) and attenuation of the X-ray beam. Hence, they represent the most promising candidate as a contrast agent for CT/X-ray imaging.

Finally, is important to notice that an intercomparison of all the nanomaterials evaluated at the same concentration [54] and using an in vivo model is quite recommended.

## 5. Conclusions

Nanomaterials are an important class of compounds with special features, including small volume, low toxicity (in many cases), and high surface area, among several others. The applicability of nanomaterials in the biomedical field has been increasing exponentially in the last few years, precisely due to the unique characteristics of these materials. Our results demonstrated that most of the nanomaterials evaluated may be used as contrast agents for both conventional X-ray and Computed Tomography. Among them, the most prominent two were hydroxyapatite microspheres (HA microspheres) and hydroxyapatite thermally treated at 1000 °C (HA 01.256 CTT 1000 °C). Conversely, hydroxyapatite doped with zinc (Zn-HA 10%), and hydroxyapatite doped with strontium (Sr-HA 10%) were the worst materials. The graphene oxide doped with copper and gold nanoparticles are also viable options.

## Figures and Tables

**Table 1:** Results of the X-ray attenuation coefficient (µ) expressed in cm^−1^ and Hounsfield units for Zn-HA 10% (1), Sr-HA 10% (2), HA 282 STT (3), HA 212 500 °C (4), HA 01.256 CTT 1000 °C (5), HA microspheres (6), AuNP (7), and Cu-GO (8) samples.

Samples	µ (cm^−1^)	µ (HU)
Zn-HA 10%	0.102	−575
Sr-HA 10%	0.134	−438
HA 282 STT	0.288	203
HA 212 500 °C	0.279	168
HA 01.256 CTT 1000 °C	0.381	596
HA microspheres	0.764	2195
AuNP	0.247	34
Cu-GO	0.240	6

## Data Availability

All data will be available under request.
